# The EANM guideline for radiosynoviorthesis

**DOI:** 10.1007/s00259-021-05541-7

**Published:** 2021-10-20

**Authors:** W.U. Kampen, B. Boddenberg-Pätzold, M. Fischer, M. Gabriel, R. Klett, M. Konijnenberg, E. Kresnik, H. Lellouche, F. Paycha, L. Terslev, C. Turkmen, F. van der Zant, L. Antunovic, E. Panagiotidis, G. Gnanasegaran, T. Kuwert, T. Van den Wyngaert, the EANM Bone & Joint Committee, the Dosimetry Committee, the Oncology & Theranostics Committee

**Affiliations:** 1 Nuklearmedizin Spitalerhof, Radiologische Allianz, Spitalerstraße 8, 20095 Hamburg, Germany; 2 Praxis NURAMED West, Max-Planck-Straße 27a, 50858 Köln, Germany; 3 Praxis Für Radiologie Und Nuklearmedizin, Friedrich-Ebert-Straße 50, 34117 Kassel, Germany; 4 Institute of Nuclear Medicine and Endocrinology, Kepler University Hospital Linz GmbH, Medical Faculty, Johannes Kepler University Linz, Altenberger Strasse 69, 4040 Linz and Krankenhausstrasse 9, 4020 Linz, Austria; 5 ÜBAG Für Nuklearmedizin, Hanau-Frankfurt-Offenbach-Gießen, Standort Gießen, Paul-Zipp-Str. 171-173, 35398 Gießen, Germany; 6 Erasmus MC, Nucleaire geneeskunde, Dr. Molewaterplein 40, 3015 GD Rotterdam, Netherlands; 7 Privatklinik Villach, Institut Für Nuklearmedizin, Dr.-Walter-Hochsteinerstrasse 4, 9504 Warmbad Villach, Austria; 8 Unité Rhumatologique de Affections de La Main, Centre Viggo Petersen, Hôpital Lariboisiere, 2 rue Ambroise Paré, 75010 Paris, France; 9 Institut de Rhumatologie Interventionnelle, 13 rue Thouin, 75005 Paris, France; 10 Service de Médecine Nucléaire, Hôpital Lariboisière, Assistance Publique- Hôpitaux de Paris, 2 rue Ambroise Paré, 75010 Paris, France; 11 Center for Rheumatology and Spine Diseases, Copenhagen University Hospital, Rigshospitalet, Valdemar Hansens Vej 17, 2600 Glostrup, Denmark; 12 Department of Nuclear Medicine, Istanbul Medical Faculty, Istanbul University, Istanbul, 34390 Turkey; 13 Nucleaire Geneeskunde, Noordwest Ziekenhuisgroep, Postbus 501, 1800 AM Alkmaar, Netherlands; 14 Diagnostic Imaging Department, IRCCS Humanitas Research Hospital, Via Manzoni 56, Milan 20089 Rozzano, Italy; 15 Department of Nuclear Medicine, Oncology Center ‘Theageneio’, Al Symeonidis 2 str, P.C 54007 Thessaloniki, Greece; 16 Department of Nuclear Medicine, Royal Free London NHS Foundation Trust, London, UK; 17 Clinic of Nuclear Medicine, Friedrich-Alexander-University, Erlangen-Nürnberg, Erlangen, Germany; 18 Antwerp University Hospital, Drie Eikenstraat 655, 2650 Edegem, Belgium; 19 Molecular Imaging Center Antwerp (MICA - IPPON), Faculty of Medicine and Health Sciences, University of Antwerp, Wilrijk, Belgium

**Keywords:** Synovitis, Arthritis, Radiosynoviorthesis

## Abstract

**Purpose:**

Radiosynoviorthesis (RSO) using the intraarticular application of beta-particle emitting radiocolloids has for decades been used for the local treatment of inflammatory joint diseases. The injected radiopharmaceuticals are phagocytized by the superficial macrophages of the synovial membrane, resulting in sclerosis and fibrosis of the formerly inflamed tissue, finally leading to reduced joint effusion and alleviation of joint pain.

**Methods:**

The European Association of Nuclear Medicine (EANM) has written and approved these guidelines in tight collaboration with an international team of clinical experts, including rheumatologists. Besides clinical and procedural aspects, different national legislative issues, dosimetric considerations, possible complications, and side effects are addressed.

**Conclusion:**

These guidelines will assist nuclear medicine physicians in performing radiosynoviorthesis. Since there are differences regarding the radiopharmaceuticals approved for RSO and the official indications between several European countries, this guideline can only give a framework that must be adopted individually.

## Introduction

Intraarticular therapy using colloidal beta-emitting radionuclides, radiosynoviorthesis or RSO, is known for almost 70 years and is indicated in patients suffering from various inflammatory joint diseases [1]. The presence of chronic synovitis needs to be confirmed before performing RSO. Three-phase bone scintigraphy, MRI, ultrasound, or histology after surgical synovectomy (e.g., in patients suffering from intraarticular diffuse-type giant cell tumor/pigmented villonodular synovitis) can be used for this purpose. The term “radiosynovectomy,” although frequently found in the literature, should not be used because the synovial membrane is not “resected” (which is meant by the Greek word “ectome”) by injection of a radiocolloid.

The mechanism of action of RSO starts with phagocytosis of the colloidal radiopharmaceuticals by the superficial lining cells of the inflamed synovial membrane [2]. The three approved radiopharmaceuticals [^90^Y]yttrium citrate, [^186^Re]rhenium sulfide, and [^169^Er]erbium citrate deliver their high-energy beta particles to the innermost cell layer of the synovium, leading to pronounced cell death, obliteration of capillary blood supply, and hence fibrosis and sclerosis of the synovial membrane (Table 1).

**Table 1:** Characteristics of the radionuclides used for radiosynoviorthesis in routine clinical practice throughout Europe (others may be used in clinical trials, not mentioned here)

	**erbium-169**	**rhenium-186**	**yttrium-90**
Phys. Half-life (hrs)	225.4	89.25	64.1
Radiation (%)	Beta (> 99) Gamma (0.1)	Beta (92.5) Gamma (7.5)	Beta (100)
Maximum beta energy	0.34 MeV	0.98 MeV	2.26 MeV
Gamma energy	8,4 keV	137 keV	–
Mean range	0.3 mm	1.2 mm	3.6 mm

These effects result in a significant decrease in inflammatory activity with less joint effusion and pain, leading to improved mobility of the joint and a better quality of life for the patient to perform daily duties and responsibilities without severe pain and pain-related restriction [3].

Besides primary inflammatory conditions, RSO is also highly effective in patients with hemophiliac joint disease, characterized by synovitis induced by iron and inflammatory factors after joint bleeding. Moreover, the inhibitory effect on neoangiogenesis may contribute to a decreased bleeding tendency and reduce the risk of developing severe joint degeneration, the so-called hemarthropathy [4].

The best clinical improvement is seen in patients suffering from high inflammatory activity in an early phase of their underlying disease when subsequent degenerative changes are not too pronounced [5]. Thus, radiosynoviorthesis should be considered early by an interdisciplinary team consisting of the referring physician (e.g., rheumatologist, orthopedic surgeon) and the nuclear medicine physician.

Side effects or complications after RSO are quite rare, besides non-serious side effects like transient radiogenic synovitis with recurrent effusion or a flush from the co-injected glucocorticosteroid. The probability of a serious adverse event (e.g., intraarticular infection or radiogenic tissue necrosis) is below 0.1 per 1000 [6]. The radiogenic induction of a malignant tumor after RSO has never been described, even after long-term follow-up [7]. Thus, radiosynoviorthesis is one local treatment option in the large armamentarium of different therapies for patients suffering from chronic synovitis.

This text updates and replaces the 2003 EANM guideline on radiosynoviorthesis and contains general information regarding the different indications for radiosynoviorthesis and contraindications, necessary pre-therapeutic diagnostics, and the treatment procedure itself. Moreover, essential aspects of this therapy, like dosimetry, radiation burden, legislative issues, and possible complications and side effects are discussed [8]. Finally, the clinical role and the impact of RSO compared to other treatments for inflammatory joint diseases are reviewed.

However, this document does not claim universal validity, especially legislative issues regarding indications, approval of the different radionuclides, or radiation protection rules may vary between different countries and must be followed according to the national laws.

Radiosynoviorthesis is an important technique for treating mono- or oligoarticular synovitis, and many factors may influence its effectiveness and safety. This project aims to establish recommendations to standardize indications and procedures to improve effectiveness and safety.

## Methods

A best-evidence review of PubMed articles published until 01/09/2020 was performed to obtain efficacy outcomes of RSO in various indications. Selection criteria included English or German language studies considered to be most relevant (e.g., adult human subjects, in vivo) to the clinical application(s) in question. It is recognized that limiting the search criteria to English and German language publications may introduce bias, but this can be justified because most RSO treatments are performed in Germany. Case reports and case series with a sample size less than 10 (arbitrarily chosen) were excluded.

## Legislative aspects and approvals throughout Europe

Radiosynoviorthesis using an intraarticular injection of beta-emitting radiocolloids is a local treatment in patients suffering from inflammatory joint disease. Depending on the joint’s size, three radiocolloids with different physical properties are used: [^90^Y]yttrium citrate for the treatment of knee joints only, [^186^Re]rhenium sulfide for mid-sized joints, and [^169^Er]erbium citrate for small joints. The current official approvals and marketing authorizations in European countries are given in Table 2. The radiocolloids are also sold in other countries worldwide, based on national rules like, e.g., in Austria, Greece, or Poland, where they are delivered by a local distributor only upon specific prescription from a doctor or a hospital (status “Medical Prescription”).

**Table 2:** Approved radiopharmaceuticals for radiosynoviorthesis (in Europe)

	[^90^Y]yttrium citrate	[^186^Re]rhenium sulfide	[^169^Er]erbium citrate
**Global status**			
Approved	12 countries	6 countries	6 countries
Sold	31 countries	17 countries	15 countries
**Approval in Europe**			
Germany	X	X	X
France	X	X	X
Switzerland	X	X	X
Spain	X	X	X
Belgium	X		
Netherlands	X		
Luxembourg	X		
Portugal	X		
Norway	X		
Ireland	X		
Turkey	X	X	X
Czech Republic	X	X	X

## Indications for radiosynoviorthesis

The intraarticular application of radiocolloids for treating inflammatory joint diseases is subject to variations in national legislative issues between different countries, and efforts are urgently needed to harmonize the availability of these treatments across Europe.

### Indications documented in the Corporate Core Data Sheets (CCDS)

The Corporate Core Data Sheets with slight national adaptations form the basis for the Summary of the Product Characteristics (SPCs) regarding the prescribing information and international marketing of the radiocolloids.

- [^90^Y]yttrium citrate is indicated in adults for the therapeutic irradiation of synovial hypertrophy of the knee joint mainly for mono- or oligoarthritis of chronic inflammatory rheumatic disorders, in particular rheumatoid arthritis and in hemophilic arthropathy.
- [^186^Re]rhenium sulfide is indicated for the treatment of rheumatoid mono- or oligoarthritis involving medium-sized joints, including rheumatoid arthritis, hemophilic arthropathy, and chronic arthropathy associated with articular chondrocalcinosis.
- [^169^Er]erbium citrate is indicated for the treatment of rheumatoid mono- or oligoarthritis of small joints of hands and feet following the failure of intraarticular corticosteroid therapy or when the latter is contraindicated.

### Deviations in Germany and Switzerland

In Germany (which accounts for approximately 80% of the European market), radiosynoviorthesis is approved for the treatment of chronic synovitis with recurrent joint effusions in patients with:

- Rheumatoid arthritis
- Seronegative spondyloarthropathy (e.g., reactive arthritis, psoriatic arthritis, ankylosing spondylitis)
- Intraarticular diffuse-type giant cell tumor/pigmented villonodular synovitis (for the prevention of relapse after surgery)
- Hemophiliac arthropathy (for prevention of intraarticular hemorrhage and subsequent arthropathy)

[^169^Er]erbium citrate is approved for the first two indications only. In Switzerland, [^186^Re]rhenium sulfide is also approved to treat the knee joint.

### Treatment of synovitis is possible, but only off-label use in selected patients

Due to the mechanism of action of RSO with superficial irradiation of the inflamed synovial membrane, related pathological situations with other underlying diseases are suitable for this treatment. However, this off-label use should be performed in selected patients only, and a detailed and documented informed consent of the patient is mandatory.

- Osteoarthritis with secondary synovitis, resistant to other therapies
- Adjuvant therapy after surgical synovectomy with recurrent joint effusions
- Recurrent joint effusion after endoprosthetic joint replacement (underlying causes of prosthesis failure, such as loosening or infection must be excluded unequivocally)

Since the approval depends on the underlying disease, RSO after surgical synovectomy or endoprosthetic joint replacement in a patient with (e.g.) rheumatoid arthritis is an approved indication.

### RSO in rheumatoid arthritis

The most common type of inflammatory arthritis is rheumatoid arthritis (RA). RA is a chronic autoimmune disease and is primarily considered to be an inflammatory joint disease. Although there are a variety of extra-articular manifestations, the pathophysiology of RA is multifactorial, resulting from genetic predisposition and various lifestyles and environmental factors. Long-term outcomes can be undesirable, involving disability and reduced quality of life, generating considerable healthcare systems costs.

#### Incidence and prevalence

RA affects approximately 0.24 to 1% of the population and is twice as common in women as men, with a typically higher prevalence of 0.5–1% in the USA and northern Europe [9–11]. The annual incidence in the USA and northern Europe is about 40 per 100,000 people [11, 12]. The lifetime risk of developing RA is 1.7% for men and 3.6% for women [13]. However, the incidence and prevalence can be up to 10 times higher for some populations, like the Pima Native Americans [14].

#### Treatment and management

Optimal care of RA patients includes both non-pharmacological and pharmacological therapies. Non-pharmacological therapies consist of diet, physical therapy, counseling, stress reduction, and surgery. Pharmacological therapies include nonsteroidal anti-inflammatory drugs (NSAIDs), systemic or intraarticular glucocorticosteroids (GC), and nonbiological and biological disease-modifying anti-rheumatic drugs (DMARDs). Since much of the joint damage occurs in the disease’s initial phases, early therapy with DMARDs has become the standard of care [15–21].

Radiosynoviorthesis (RSO) has been used to treat synovitis for more than half a century. Radiation of the synovium causes synoviocyte and inflammatory cell necrosis and inhibited cell proliferation, temporarily improving synovitis [22]. In 1952, the Austrian Fellinger was the first to apply RSO in RA with a colloidal solution of a gold radioisotope [1]. In Europe, the most used radiopharmaceuticals are [^169^Er]erbium citrate, [^186^Re]rhenium sulfide, and [^90^Y]yttrium citrate [8]. Besides these radiopharmaceuticals, a range of other isotopes has been used for RSO like dysprosium-165, holmium-166, lutetium-177, phosphorus-32, rhenium-188, samarium-153, and gold-198 in different colloidal preparations [23–29]. Co-administration of a glucocorticosteroid (e.g., triamcinolone acetonide or triamcinolone hexacetonide) helps bridge the lag phase between injecting the radiopharmaceutical and the onset of the effect of RSO. It also reduces the risk of radiation-induced synovitis and the severity of hypervascularity and hyperpermeability that may cause leakage from the joint. Table 3 shows commonly used activities of the radioisotopes and doses of triamcinolone acetonide for various joints.

**Table 3:** Activities of the radiocolloids and doses of triamcinolone acetonide (TA) for various joints

Joints	[^90^Y]yttrium citrate (MBq) and TA (mg)	[^186^Re]rhenium sulfide (MBq) and TA (mg)	[^169^Er]erbium citrate (MBq) and TA (mg)
Knee Re-RSO	185–222 / 40 111–222 / 40		
Shoulder		74–148 / 40	
Elbow		74–111 / 40	
Wrist		37–74 / 20	
Hip		74–148 / 40	
Ankle		74 / 40	
Subtalar		74 / 20	
*Knee (in CH)		110–185 / 40	
CMC I / SIJ			20–80 / 8
MCP others			20–40 / 8
PIP / SCJ			10–20 / 4
DIP			10–15 / 4
MTP			30–40 / 8
TMT			20–40 / 8
ACJ / TMJ			20–40 / 4

Abbreviations: CMC = carpometacarpal joint, MCP = metacarpophalangeal joint, SIJ = sacroiliac joint, PIP = proximal interphalangeal joint, SCJ = sternoclavicular joint, DIP = distal interphalangeal joint, MTP = metatarsophalangeal joint, TMT = tarsometatarsal joint, ACJ = acromioclavicular joint, TMJ = temporomandibular joint

*[^186^Re]rhenium colloid is additionally approved in Switzerland for the treatment of the knee joint in patients younger than 20 years and with only a slight synovial thickness.

In the majority of published literature, the effectiveness of RSO is based upon the improvement of patient-reported outcomes like pain on a visual analog scale (VAS), joint swelling, and range of motion. Besides these improvements, the effect of RSO has been monitored with imaging modalities, including three-phase bone scintigraphy, ultrasound (US), and magnetic resonance imaging (MRI) [30–32]. A decline in the erythrocyte sedimentation rate (ESR) and serum levels of C-reactive protein (CRP) after RSO has also been reported [33].

Several reviews with and without meta-analyses have been published [34–39]. Jones et al. concluded that [^90^Y]yttrium colloid was superior to placebo administration (odds ratio (OR) 2.42, 95% confidence interval (CI) 1.02–5.73) for achieving treatment success, but possible publication bias could not be ruled out. [^90^Y]yttrium colloid was not superior to triamcinolone (OR 1.89, 95% CI 0.81–10.55) [37]. Deutsch et al. concluded that RSO is efficacious in controlling rheumatoid arthritis symptoms, even considering the paucity of well-controlled trials and rigorous clinical follow-up [34]. The overall response rate in RA varied between 35 and 100%, with better responses in joints with less joint damage. In contrast, Heuft-Dorenbosch et al. stated: “From the point of view of evidence-based medicine it should be seriously questioned whether [^90^Y]yttrium colloid synovectomy deserves a place in clinical practice” [36]. However, this was based on two trials consisting of only 23 and 22 RSO procedures. Kresnik et al. found a mean improvement rate of 67 ± 15% in RA in clinical outcome parameters. Interestingly, RSO was successful in 73 ± 12% and 64 ± 17% for Steinbrocker I and II joints, respectively, compared to a mean success rate of only 52 ± 23% for Steinbrocker III and IV joints [5]. Klett et al. concluded that there is good evidence of efficacy for RSO of medium-sized joints in RA using [^186^Re]rhenium colloid and that it is a suitable second-line treatment for RA patients in whom other therapies (including GC injections) have failed, with success rates varying between 34 and 94% [38]. Van der Zant et al. reported a pooled OR to achieve treatment success of 4 (95% CI 1.2–14) for RSO of the knee compared to intraarticular GC injection or saline at 6 months, and an OR of 1.7 (95% CI 0.69–4) at 12 months. The ORs of treatment success for RSO with [^186^Re]rhenium colloid and [^169^Er]erbium colloid combined were 2 (95% CI 0.66–6) and 2 (95% CI 1.09–3.5) at 6 and 12 months, respectively [39].

In the last decade, several studies on the effect of RSO in RA have been published, mostly consisting of case series. One controlled, randomized, and double-blinded trial compared [^90^Y]yttrium colloid + GC and [^153^Sm]samarium colloid + GC to GC alone (control group). Only for the pain parameter, [^90^Y]yttrium colloid + GC proved superior to [^153^Sm]samarium colloid + GC and GC at 1 week, and [^90^Y]yttrium colloid + GC proved to be superior to GC at 48 weeks. Furthermore, the study concluded that there is no indication for RSO of the knee with [^153^Sm]samarium colloid in RA patients [35]. Gonçalves et al. published data on ultrasound-guided RSO of the knee using [^90^Y]yttrium colloid as well. Of the studied patients, 11 had RA. At 6 months, there was an improvement in swelling, tenderness, and pain. No short-term side effects or complications were recorded [40]. Goetz et al. published long-term results of combined arthroscopic and radiosynoviorthesis of the knee in RA patients. The combined treatment led to stable improvement for at least 5 years, but surgical re-interventions were needed at 14 years of follow-up in 16 of 38 patients within mean 5.7 years (range 1.2–9.5 years) [41]. Wong et al. concluded that RSO with [^90^Y]yttrium colloid remains a safe and effective treatment in an era of improved DMARDs. The clinical response in 46 RA knee joints was mild, moderate, and complete in 24%, 26%, and 28% at 3 months, respectively. However, at 36 months, the response declined, especially in mild responders [42]. The study by Zalewska et al., including 34 RA patients, found the most significant decline of synovial hypertrophy and improvement of inflammatory parameters in RA patients [33]. Kim et al. using [^90^Y]yttrium hydroxyapatite found a reduction of pain, swelling, and improvement of knee motion in approximately 80% at 6 months and 76% at 12 months [43]. In a study by Miszczyk et al. of 394 RSOs of the knee with [^90^Y]yttrium colloid, including 73 RA cases, pain relief was observed in 81% at 6 months (33% with complete pain relief) and in 87% at 1 year. The likelihood of pain relapse in RA cases was 15% at 6 months and 27% at 12 months [44]. In a multicenter study by Liepe et al., 99 knee RSOs (68 with [^90^Y]yttrium colloid, 15 with [^32^P]phosphorus colloid, and 16 with [^188^Re]rhenium colloid) were compared with 46 intraarticular GC injections. Pain relief at 3 months was 86% for the RSO group versus 67% for the GC group. At 6 and 12 months, the numbers were 72% versus 46% and 46% versus 21%, respectively. There was no significant difference between the three radiocolloids [45]. A prospective study by Amini et al. analyzing 23 RSOs of the knee using [^32^P]phosphorus colloid in RA patients reported excellent response in 56%, moderate response in 9%, and poor response in 35% [46]. Shito et al. reported a success rate of 80% at 6 months after RSO of the knee in RA patients with [^177^Lu]lutetium hydroxyapatite [47]. In a retrospective study of RSO, using [^169^Er]erbium, [^186^Re]rhenium, and [^90^Y]yttrium colloid, in 577 joints of 137 RA patients, Liepe et al. reported success rates (excellent or good response) in 57% of the treated knees, 63% of shoulders, 60% of wrists, 64% of ankles, 54% of thumb bases, 55% of MCPs, 54% of PIPs, 53% of DIPs, and 54% of MTPs [48].

As RA is a systemic disease, it is recommended to start with anti-inflammatory drugs with or without DMARDs. Persistent synovitis of one or more joints could be treated with intraarticular GC injections, and RSO is indicated after at least one unsuccessful intraarticular GC injection in RA patients treated with DMARDs. The best results for RSO are reported for joints with minimal or moderate joint damage, and RSO should therefore be considered early in RA in case of persistent synovitis [5]. Contraindications for RSO are listed in Table 4.

**Table 4:** Contraindications for RSO

Contraindications
Absolute:
Pregnancy Breastfeeding Local skin infection or septic arthritis Ruptured popliteal cyst Recent joint surgery or arthroplasty with fresh surgical scars (< 6 weeks) Uncontrolled bleeding (including massive hemarthrosis)*
Relative:
Extensive joint instability with bone destruction High-grade bone destruction For children and young patients (age < 20 years old), RSO should be a restricted indication and the benefit of RSO should clearly outweigh the potential hazards, compared to possible alternative treatments

*Except for hemophiliac patients, see dedicated section.

### RSO in hemophilia

#### Pathogenesis of synovitis in hemophilia or hemarthropathy

Hemophilia is a bleeding disorder due to an X-linked inherited deficiency or absence of coagulation factors. As a result, affected patients experience bleeding mostly in the musculoskeletal system, especially in joints (80–90%) [4], causing synovitis. The pathophysiology is mainly driven by an inflammatory response to the iron load in the joint. The resulting neoangiogenesis causes an increased vulnerability of the synovial membrane and, consequently, an increased tendency to bleed [49, 50].

Acute synovitis is treated by clotting factor substitution, but if this condition persists in time, the patient will develop chronic synovitis with cartilage and bone changes and subsequent hemarthropathy [51].

The vicious circle of bleeding—synovitis—neoangiogenesis—increased bleeding tendency leads in the long term to fibrosis, cartilage and bone destruction, and ultimately spontaneous arthrodesis, which can lead to a severe restriction of daily activities even in childhood [52].

In hemophilia, bleeding can occur in any joint. Nevertheless, in many patients, one joint is more affected than the other (the so-called target joint). If not treated in a sufficient manner until all symptoms have subsided, the recurrent bleedings do not leave time for complete healing, leading to the vicious circle described above and ultimately leading to hemarthropathy [53].

In patients with chronic synovitis (longer than 3–6 months), RSO reduces synovitis symptoms by inducing fibrosis of the synovial surface and decreasing the bleeding tendency [54–56].

#### Diagnosis of synovitis in pediatric and adult hemophilia

The most commonly affected joints in hemophilia patients are the knee, ankle, and elbow joint [57]. RSO is indicated if synovitis can be demonstrated or three joint bleedings occur within 6 months, despite clotting factor substitution [55, 58, 59].

In contrast to other diseases, synovitis in hemophilia can persist longer with only mild clinical symptoms, so-called silent symptoms, especially with prophylactic factor substitution. Chronic synovitis may develop from minor injuries with recurrent micro-bleeding and finally damage to the cartilage and bone. Thus, due to this sometimes heavily protracted course of chronic synovitis, pre-therapeutic imaging may be older than 4–6 months in patients with hemophilia [50, 60–62], compared to the need of more current imaging in acute synovial inflammatory diseases.

The diagnosis of synovitis is based on clinical evaluation and adequate imaging, including ultrasonography, MRI, or multi-phase bone scintigraphy [58, 60].

For children, radiation-free methods should be preferred. However, with adequate clotting factor substitution, synovitis is often only very discreet and difficult to visualize with any available method [58]. Ultrasonography is used for initial joint assessment but also to evaluate disease progression. The disadvantage is that this procedure is operator-dependent.

For the diagnosis of hemophilia-related synovitis, an ultrasonography protocol was developed (HEAD-US Score) that allows the synovia and cartilage to be assessed even by inexperienced examiners. Following this protocol makes it possible to detect synovitis with a high degree of probability. Nevertheless, it is unclear whether this finding alone is sufficient as an indication for RSO [63–65].

MRI is the reference standard for detecting synovitis and early degenerative changes, but it is frequently only possible with sedation in young children. The use of contrast agents (e.g., gadolinium) is reserved for cases with mild signs of synovitis. The discussion of possible long-term side effects of these contrast agents due to basal ganglia deposition, recently resulting in a “precautionary approach” by the EMA, may be overcome by the use of macrocyclic agents [58, 66, 67].

Multi-phase bone scintigraphy can detect synovitis in patients with hemophilia; however, treatment with clotting factors may normalize the articular perfusion on blood pool images in some patients with painful joints. However, even in patients with normal findings on bone scintigraphy, it has been shown that they can still benefit from the RSO [68].

#### Efficacy of RSO in hemophilia

Seventy to 90% of patients benefit from RSO concerning bleeding frequency, the intensity of pain, joint function, and thickness of the synovium [4, 50, 53, 56, 58, 69]. The primary aim of RSO, namely to stop bleeding, is usually reached. For example, in 2002, Manco-Johnson et al. described a complete cessation of bleeding after RSO with [^32^P]phosphorus colloid [70]; Kavakli et al. reported that bleeding was successfully treated in more than 80% of the patients [71]. The same results were shown in the study of de la Corte-Rodriguez et al., who investigated ten parameters after RSO. All parameters (bleeding frequency, pain, joint function, ROM, synovitis thickness, clinical scores, etc.) improved, some statistically significant, without assessing bone damage by X-ray. Synovial thickness decreased as seen in sonography or MRI [72].

The improvement of mobility is limited to cases without established permanent joint damage at the time of treatment. However, some studies have not shown improvements in the range of motion (ROM) of the treated joint [54].

The best results are achieved before the onset of hemarthropathy and are best in ankle joints, followed by elbows and knee joints [71, 73]. A more recent study suggests that RSO slows the development of hemarthropathy in younger patients, but it remains uncertain whether RSO prevents hemarthropathy [74].

Therefore, RSO should be used as early as possible before developing hemarthropathy [55, 58]. The success of the procedure varies depending on the joint and the severity of the hemarthropathy. Two recent studies show that the dosage of coagulation factors needed decreases after RSO [75, 76]. In the study by Kachooei et al., [^188^Re]rhenium colloid was used, but similar results can be expected with the approved [^186^Re]rhenium colloid.

In pediatric hemophilia, RSO is indicated when chronic persistent or recurrent synovitis with or without recurrent joint bleeding (2–3 episodes per 6 months) is detectable. It can also be used when moderate hemarthropathy exists and surgical interventions are not yet required. RSO is an option when inhibitors against factor VIII/IX are present [77, 78] or when synovitis persists over 6 months despite intensified factor therapy [58, 78]. Before the onset of hemarthropathy, RSO should be used as a first-line treatment in chronic synovitis [58, 78–80]. Finally, if the synovitis persists or relapses after the first RSO, it is proposed that the procedure can be repeated up to three times. If synovitis persists after the third RSO, it should be treated surgically [58, 78, 81].

### RSO in osteoarthritis

Similar to rheumatoid arthritis, RSO could be helpful in selected patients with osteoarthritis and proven synovitis, resistant to other therapies; however, due to national rules, it has to be done as an off-label use in some countries.

Osteoarthritis (OA), also known as degenerative joint disease, is a large and increasing burden for the health systems in many countries. In Europe, more than 30 million people might be affected. Similar data with an increased prevalence of osteoarthritis is also seen worldwide in an aging population [82]. Besides simple mechanical reasons for the induction of degenerative joint disease, several other risk factors like genetics, sex and race, metabolic syndrome, mechanical injury and overuse, and secretion of pro-inflammatory cytokines inside the joint may lead to synovial inflammation and initial cartilage defects and may precede subchondral bone destruction.

Most often, the weight-bearing joints of the lower extremities are affected. In finger OA, the distal interphalangeal (DIP) joints, proximal interphalangeal joints (PIP), and the first carpometacarpal (CMC) joints are frequently involved.

Symptoms include joint pain, stiffness, and sometimes a joint effusion. Joint pain is the leading clinical symptom of OA. It was discussed in the literature that the intensity of the pain depends on the radiological degree of bone alterations in the affected joint. Additionally, recent animal studies demonstrated that blood vessels with nociceptive nerve fibers penetrating the osteochondral junction into the cartilage might contribute to the joint pain [71]. Furthermore, synovitis has been reported as the determining factor for neuronal sensitization and the progression of osteoarthritis. It has also become evident that the synovial membrane’s inflammatory process precedes the detection of degenerative cartilage changes. The inflamed synovial membrane produces inflammatory cytokines that cause cartilage damage [70, 83]. Therefore, prompt treatment of synovitis is recommended to improve pain and prevent disease progression [84–86].

In the literature, the improvement rate after RSO ranges from 40 to 89%. In a recent long-term prospective study of RSO in synovitis in OA of the knee joint, Szentesi et al. [87] reported that in 69 patients with early-stage disease (Kellgren-Lawrence grade I/II), an excellent/good response to pain, joint mobility, and function was observed in 82.5% for 1 year and 73.3% for 8 years after therapy. Even in grade III patients (*n* = 72), an excellent/good response was observed in 45.9% and 41.2% after 8 years.

In a meta-analysis of 121 patients with OA treated with RSO, the mean success rate was 56 ± 11% [5]. The observation period was 1 year in this analysis. Furthermore, it could be demonstrated that the improvement rate depended on pre-existing degenerative morphological changes. The improvement rate was > 80% when there were no degenerative changes and 60–80% in case of moderate changes. However, the response rate decreased in severe degenerative changes, but it was still classified as “helpful” with a success rate of < 60%.

Similar results were also found by other authors. In patients with OA of the knee, the overall success rate in pain was 86%, and knee flexibility was improved in 65%. Furthermore, the clinical improvement was inversely related to radiographic knee damage, patient’s age, and duration of the disease [88, 89]. These results suggest that the therapeutic effect depends on the underlying disease and the type of joint.

Zuderman et al. reported that the success rate of RSO for the small-, medium-, and large-sized joints were 89%, 86%, and 79%, respectively [90]. If the underlying diseases were compared with each other, irrespective of the different joints for RA and OA, the success rates were 89% and 79%, respectively, after 12 months [90]. For large joints, Rau et al. reported that the clinical outcome in a multicenter study of RSO in various joint disorders was significantly better for OA. The response rate for small- and large-sized joints in OA was similar to patients with RA [91]. However, in a study by Kampen et al., the authors found that the therapy was also highly effective in digital joint OA with local synovitis [92]. The best results were obtained in the thumb base joints, whereas distal interphalangeal joints are less likely to respond. All patients also reported an improvement in their manual activities.

Another group performed a double-blind controlled prospective study on 22 patients with local synovitis in OA of the thumb base joints. The effect of [^169^Er]erbium colloid combined with GC was compared to GC injection alone with 1 year of follow-up. RSO combined with GC resulted in significant reductions of pain, inflammation, and improvement of motion.

In a randomized, double-blind, placebo-controlled study of RSO of the upper extremity joints in 44 patients, the response rates after 6 and 12 months were 69% for RSO plus GC and 29% and 32% for placebo plus GC injection. The radionuclide plus GC showed a significantly better response than GC plus placebo [93]. The authors advocate RSO over the sole use of GC intraarticular injection in the finger, ankle, and wrist joints.

RSO can also prevent deterioration of inflammatory and radiographic features even in patients suffering from osteoarthritis. In a recent study by Szerb et al., it was reported that among 48 patients with osteoarthritis of the hip joints and 43 patients suffering from OA in ankle joints, RSO could prevent radiologic deterioration among 70.6% of the hip patients and in 79.1% of the treated ankle joint patients. The mean follow-up time was 9.2 years [94].

In another study from the same group, including 207 treated knee joints, 163 had the same Kellgren-Lawrence grade at the time of follow-up compared to baseline, while 44 had deteriorated. The mean duration of follow-up was 8.7 years. RSO prevents radiological progression in 79% of the treated joints [95].

As an alternative to RSO, GC intraarticular injections can reduce inflammation and pain for a limited time, but this effect is usually transient with subsequent disease progression. Other authors also reported similar results, with pain relief after intraarticular GC injection lasting about 3–4 weeks [73, 96]. Several authors reported the efficacy of intraarticular GC therapies. In a systemic review, Wernecke et al. showed a chondrotoxic effect of triamcinolone in vitro, whereas in vivo studies demonstrated cartilage protection with a low-dose GC injection. These results were also supported by Raynold et al., concluding that the effect of GC may be dose- and time-dependent. Treatment with a low dose for a short time may be beneficial, whereas high-dose and long-term treatment could be harmful [97, 98]. However, data from a Cochrane systematic review showed that the effect of intraarticular GC for knee osteoarthritis does not persist after 6 months [99].

#### Efficacy of RSO in knee arthroplasty

RSO was also studied for treating recurrent joint effusions after knee arthroplasty. However, only scarce data are available in the literature. The initial results with RSO in knee arthroplasty were reported by Mödder et al. [100]. In their study, 107 patients with chronic joint effusion due to polyethylene disease caused by abrasion particles from the polyethylene inlays were treated with [^90^Y]yttrium citrate. In 93/107 (87%) of patients, the joint effusion resolved entirely after therapy.

In a study by Mayer-Wagner et al., 55 patients with chronic joint effusion after knee arthroplasty were treated with radiosynoviorthesis using [^90^Y]yttrium colloid [101]. Significant improvements in pain, effusion, and function were seen in 54% of patients. For most of the patients in whom RSO treatment failed, complications of the arthroplasty like infection, loosening, allergy, and trauma were detected. Thus, all these underlying causes of prosthesis failure must be excluded unequivocally prior to RSO using professional clinical examination, laboratory testing of inflammatory blood values, synovial fluid aspiration, and appropriate imaging.

#### Efficacy of combined surgical synovectomy and RSO

Surgical synovectomy is well established in the local treatment of synovitis. However, due to the surgical trauma and incomplete removal of all pathological tissue with minimal arthroscopic synovectomy, the recurrence rate is high and amounts to 30% with long-term follow-up [83, 102]. Therefore, several authors have studied the usefulness of the combination of arthroscopic subtotal synovectomy and radiosynovectomy.

In one study by Akmese et al., the authors compared combined arthroscopic synovectomy and RSO to treat chronic non-specific synovitis of the knee [103]. They found significant improvements in the range of motion and severity of joint effusion. Also, pain and synovial membrane thickness were significantly reduced (82% and 54%, respectively). Clinically and radiologically, using MRI, there was no recurrence after 3 years.

Similar results were also found by other authors. Kerschbaumer et al. reported significantly better long-term clinical results (8 years) in 141 knee joints treated with the combination therapy compared to patients receiving RSO alone [104]. Additionally, open synovectomy is preferred over arthroscopic synovectomy if tenosynovectomy is simultaneously required [105, 106].

Concerning the timing of RSO relative to the surgical procedure, it was suggested to perform RSO 6 weeks after surgery to allow the postoperative edema to diminish. Also, healing of the surgical wound is almost complete by that time, preventing any leakage of the injected radionuclide. Moreover, postoperative inflammation will have peaked, improving the efficacy of the anti-inflammatory effect of RSO [103].

## Summary of RSO indications

RSO is effective in controlling symptoms of persistent synovitis in RA patients. However, the relief of symptoms declines over time. Also, RSO provides acceptable clinical results in selected patients with OA according to the severity of degenerative changes. The co-injection of a GC with RSO provides favorable clinical results and is preferred in clinical routine. In arthroscopic synovectomy, the combination with RSO provides significantly better clinical results than surgery alone and the recurrence rate of diffuse-type giant cell tumor/pigmented villonodular synovitis is reduced by performing an RSO after surgical synovectomy.

The treatment of synovitis in hemophilia is supported by the most recent guidelines from major organizations [4, 58, 69]. It is a highly effective treatment with few side effects, and RSO should be used early in chronic synovitis in hemophilia. RSO leads to a reduction of bleeding frequency and slows the development of symptoms related to synovitis and hemarthropathy. The considerable benefits of the procedure outweigh the potential radiation risks. Therefore, RSO should also be considered a treatment option in childhood, if indicated.

## Pre-therapeutic diagnosis of synovitis

The aim of the pre-therapeutic assessment is the confirmation of synovitis and the exclusion of contraindications against RSO.

### Medical history and clinical assessment

A detailed medical history will help ascertain the appropriateness of RSO with respect to the underlying disease. Both the presence of local (e.g., hot or swollen joints, pain, morning stiffness) and constitutional symptoms (e.g., high-grade fever, weight loss, malaise, muscle weakness, rash, adenopathy, ulcers, dry eyes) should be checked as part of the work-up of synovitis to determine the underlying condition. The swelling of joints needs to be documented according to number (single or multiple), pattern (size, symmetry), and severity. Pain characteristics should be detailed: the pain quality, time of onset, eliciting or remitting factors, severity (e.g., documentation by a VAS), and duration.

### Multi-phase bone scintigraphy

Synovitis can be visualized by three-phase bone scintigraphy using radiolabeled bisphosphonates. In particular, in the early-phase images of the affected joints acquired approximately 3–10 min after IV injection of the tracer, very typical synovitis features in the affected joints can be determined, and a polyarticular involvement can be documented or even ruled out [101]. In contrast, late-phase acquisitions 2 h after tracer injection demonstrate osseous involvement. Multi-phase bone scintigraphy can also show signs of inflammation in joints that are clinically asymptomatic and provide insight into the distribution of affected joints and the underlying condition [107]. A supplementary SPECT/CT of the affected joint(s) may allow detecting bone or cartilage damage (e.g., osteochondrosis dissecans) by a more precise localization of tracer uptake [108].

To avoid unnecessary radiation exposure, bone scintigraphy should only be performed in patients suspected of polyarticular disease and/or if no other meaningful proof of synovitis (e.g., other imaging done so far, histological diagnosis after surgery) is already available. This holds especially true for children.

### Joint ultrasound (if possible with Doppler)

**Fig. 1:**
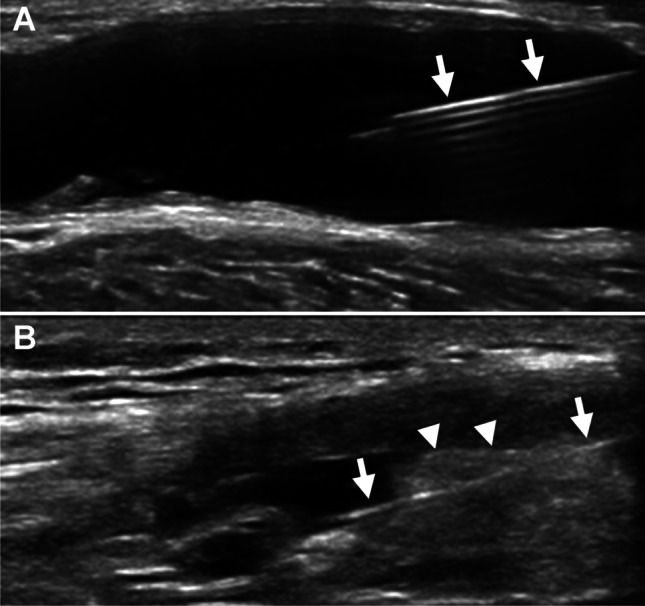
Ultrasound of (A) the puncture of a large Baker’s cyst and (B) the puncture of a knee joint from lateral into the suprapatellar recess with the whole needle in view (arrows), here penetrating a thick synovial fold (arrowheads) (images courtesy of Dr. B. Boddenberg-Pätzold, Cologne/Germany)

Joint ultrasound is suitable for assessing joint effusion, adhesions or septae, synovial morphology (e.g., presence of a coral reef-like mass, rotator cuff rupture), periarticular structures (e.g., bursitis, tenosynovitis, enthesopathy), and perfusion. Before knee RSO, the presence of a synovial popliteal cyst (i.e., Baker’s cyst) must be assessed using ultrasound (Fig. 1). Depending on the cyst’s size and the presence of a valve mechanism, ultrasound-guided relief of the cyst by fluid aspiration can be considered to avoid rupture after RSO. A pre-existing rupture of a cyst must be ruled out before RSO because of the risk of leakage of radioactivity into the surrounding soft tissues. Assessment of synovitis using ultrasound with Doppler can also be considered [109], an experienced examiner assumed.

### MRI (with or without IV contrast)

MRI using fluid-sensitive sequences, perhaps with the administration of contrast agents, is also suitable to document synovitis. Moreover, it can help detect other synovial changes, cyst formation (including Baker’s cyst), surrounding soft tissue changes, and possible joint infection when clinically suspected (e.g., high protein liquid). Other causes of joint pain may be visualized, including the destruction of the subchondral border lamella with bone marrow edema or osteochondrosis dissecans. MRI should be taken into account, especially in children (e.g., hemophilia) due to the lack of radiation exposure. On the other hand, the routinely acquired protocols are used to image a single joint or at least a local region, like the hand or foot. Thus, in patients with systemic inflammatory diseases, bone scintigraphy is still the method of choice.

### X-ray or CT

A recent (maximum 4–6 months old) X-ray image in two planes of the joint to be treated or CT (to exclude other joint pathologies like fractures, bone tumors, Ahlback’s disease, free joint bodies, or severe bone destruction) should be available before RSO. Both techniques can be performed with or without the use of intraarticular contrast. X-rays are routinely performed and, therefore, are readily available in most cases. Additional X-rays, especially of small joints, can provide important information if bone scintigraphy or ultrasound shows equivocal findings requiring further clarification.

While in most cases, the pre-therapeutic diagnosis of synovitis can be ascertained using the techniques described above, the following diagnostic modalities may provide additional information in selected patients.

### Arthro-scintigraphy

This technique can also be used to exclude a rupture of Baker’s cyst of the knee in selected patients. If leakage of a radiolabelled colloid (e.g., [^99m^Tc]technetium nanocolloid) injected into the knee joint is detected in the popliteal soft tissue surrounding Baker’s cyst after movement of the joint, a rupture is assumed, and the RSO is strictly contraindicated in this patient. However, since radiocolloids are not officially approved for intraarticular application, the patient must provide informed consent that this examination is an off-label use.

### [^18^F]fluoro-deoxyglucose PET/CT ([^18^F]FDG PET/CT)

[^18^F]FDG PET/CT can be used to image inflammation because increased [^18^F]FDG uptake occurs in the presence of activated inflammatory cells. As the synovial intima contains macrophages, [^18^F]FDG PET/CT allows sensitive detection of active lesions of RA, including clinically unexpected lesions like subclinical synovitis. Moreover, a dramatic increase in [^18^F]FDG uptake in both synovial fibroblasts and macrophages has been observed when these cells were exposed to inflammatory cytokines released in RA, such as TNF alpha, IL-1, and hypoxia. [^18^F]FDG uptake assessed with the standardized uptake value (SUV) strongly correlates with pannus volume as evaluated at MRI and shows a correlation with clinical symptoms of arthritis such as tenderness and swelling [110–113].

## Procedural aspects

All applicable national regulations must be complied with regarding the approved indications. The diagnosis of synovitis and the referral for RSO are usually made in cooperation with the referring rheumatologist, orthopedic surgeon, hand surgeon, hematologist, or pediatrician. However, the specialist in nuclear medicine, doing the final intraarticular injection of the radiocolloid, is responsible for the ultimate RSO indication and is liable for RSO-related complications.

Since the activities used for radiosynoviorthesis are largely standardized, a pre-therapeutic dose calculation by a medical physicist is not routinely necessary; however, in case of deviation from standard doses, a medical physicist should be available for consultation. However, national legislation regarding the requirement of individualized dose estimations by a medical physicist should be adhered to.

### Information required before RSO

- Confirmation of the therapeutic indication. Documenting the patient’s symptoms and their intensity (e.g., VAS) is recommended before treatment to allow assessment of clinical results during follow-up.
- Documentation of previous therapies: joint punctures, intraarticular GC injections, surgical interventions (with details of the time and clinical course).
- Current medication: especially treatment with GC and anticoagulants.
- Coagulation status before the joint puncture if there is suspicion of a coagulopathy. The question if a therapy with clotting inhibitors should be interrupted before RSO is still under debate. Several studies with patients undergoing arthrocentesis or intraarticular injections during anticoagulation therapy with different agents (including vitamin K antagonists) did not show an elevated bleeding frequency [114–116]. Also, the new oral anticoagulants usually do not have to be paused at all or only on the same day as the RSO procedure [117]. Since a joint puncture has only a low risk of bleeding, discontinuation of therapy or switching to heparin is only necessary in rare cases with genetic clotting disorders or a history of recurrent thromboembolic events. Nevertheless, a careful consideration between the risk of a local bleeding complication after RSO against the risk of a thromboembolism due to the higher clotting activity should be made for every patient based on individual risk factors. The patient must be informed about his individual risk–benefit estimation and should remain under close monitoring after RSO. However, any change in treatment should be made only in consultation with the prescribing family physician or coagulation specialist.
- Current imaging (not older than 4–6 months): conventional X-ray, multi-phase skeletal scintigraphy, MRI, ultrasound (perhaps with Doppler).
- Exclusion of a ruptured Baker’s cyst.

### Informed consent

Before the RSO procedure is performed, the patient must be informed in due time about the following aspects:

- The treatment procedure and possible side effects of the intraarticular puncture and RSO (especially about a low risk of infection or of radiation-induced soft tissue necrosis from leakage out of the puncture channel or from paraarticular injection)
- Radiation burden
- Alternative treatment options
- The necessity to immobilize the joint treated with RSO (e.g., bandage, splinting) possibly requires thromboembolic prophylaxis for 48 h.
- In women of childbearing age, pregnancy should be avoided for at least 4 months after RSO (according to the SPC).
- Breastfeeding should be terminated prior to RSO. A transfer of [^90^Y]yttrium colloid into breast milk was shown [118]. There are no data yet available for [^186^Re]rhenium colloid and [^169^Er]erbium colloid; thus, the same procedure is advisable and is given in the SPC.
- In diabetic patients, the co-injection of a crystalline glucocorticoid may lead to a higher demand of insulin or oral antidiabetic drugs for roughly 2 days, following the SPCs of injectable steroids. Thus, the use of intraarticular glucocorticoids in diabetic patients is not contraindicated but an adequate information about this possible side effect is important.
- According to local regulations, a written, dated, and signed informed consent form may be required.

### Radiation protection according to local legislation

- Handling authorization for the radionuclides used
- Appropriate measures to monitor for contamination
- Storage of the nuclides and waste disposal
- Radiation protection monitoring, possibly using finger ring dosimeter with beta emitters
- X-ray for puncture under image converter control (exception: knee joint)
- Mandatory patient discharge information. In particular, including advice on the urinary radiopharmaceutical excretion during the first 2 days after administration. Patients should be advised to observe rigorous hygiene in order to avoid contaminating groups at risk using the same toilet facility, by flushing twice and observing good hand hygiene. Significantly soiled clothing should be washed separately. Incontinent patients should be catheterized prior to radiopharmaceutical administration. The catheter should remain in place for 3 to 4 days. Catheter bags should be emptied frequently. Gloves should be worn by staff caring for catheterized patients.

### Treatment rooms according to local legislation

- Treatment may only be carried out in rooms approved by the competent authority for handling open radioactive substances. In particular, if the procedure is performed outside the nuclear medicine department (e.g., operating theater).
- Intraarticular punctures may only be performed if the hygienic requirements are met. A corresponding hygiene plan must be available.
- Rooms and facilities require regular cleaning and disinfection. The number of people in the treatment room should be limited to what is necessary.

### Preparation of the patient and physician

Intraarticular injections and punctures require careful planning and execution [119]. The following procedures may need to be adapted according to local legislation:

- The patient washes the area of the puncture.
- The injection area must be exposed to such an extent that contamination by clothing is avoided, and the physician is not hindered.
- Hair that may interfere should be removed or clipped before the injection, using scissors or swabs. Shaving the hair in the injection area should be avoided because it could lead to skin injuries prone to infection.
- Hygienic disinfection of the puncture site. The injection site and its surroundings must be treated with an approved skin antiseptic. The antiseptic can be applied by spraying or wiping so that it is brought in from all sides; achieving a thorough wetting of the skin is required. The application should be performed in a centrifugal fashion, from the inside to the outside. The exposure time must be observed in accordance with the manufacturer’s instructions, usually at least 1 min. Using colored antiseptics can make it easier to identify the area being treated.
- If possible, the puncture site should be covered with a sterile perforated cloth. There is no scientific evidence for the general use of perforated sheets or other covers. A perforated sheet can be useful if, for instance, there is a risk of contamination from possible contact of the hand used for injection or the syringe or needle with the patient (e.g., pubic hair in the event of a hip puncture).
- The following applies to the physician and any assistants: clothing, especially sleeves, must not pose any risk of infection; appropriate protective clothing (e.g., apron) may be required. The following applies to the physician: hygienic hand disinfection, wearing sterile gloves and a face mask. Assistants for the puncture or injection must carry out hygienic hand disinfection and wear face masks before performing any operations to prepare the injection site
- Sterile disposable cannulas and sterile disposable syringes must be used.

### Preparation of the activity

**Fig. 2:**
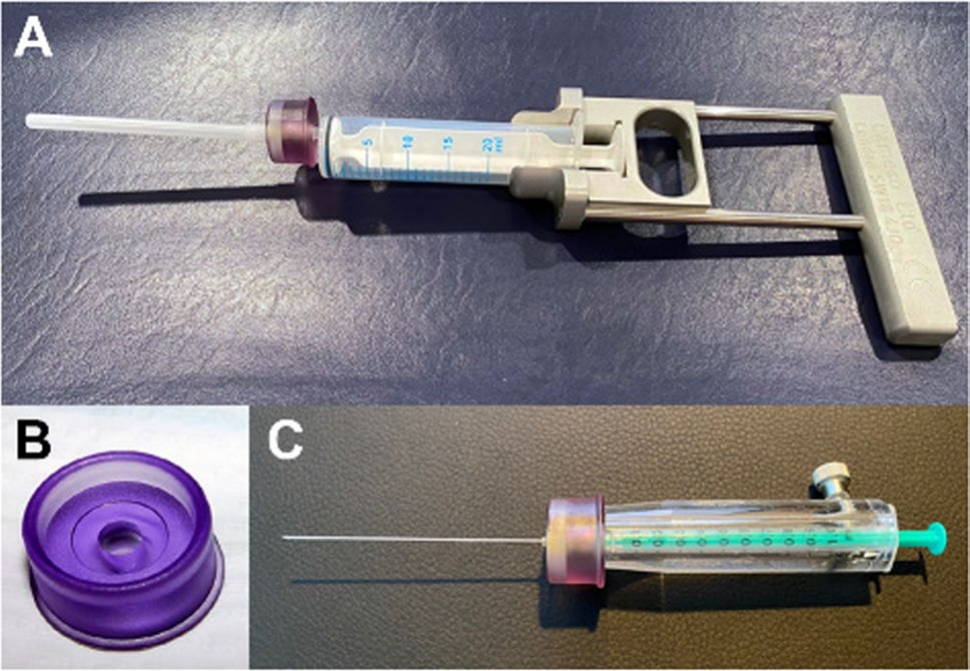
Set of instruments for the puncture of a knee joint, (A) large syringe (20 ml) with applicator for aspiration of joint effusion, (B) a ring made of acrylic plastic to reduce the local radiation dose at the base of the needle significantly, and (C) a syringe shielding made of acrylic plastic to reduce radiation load from yttrium-90 and rhenium-186 during RSO

**Fig. 3:**
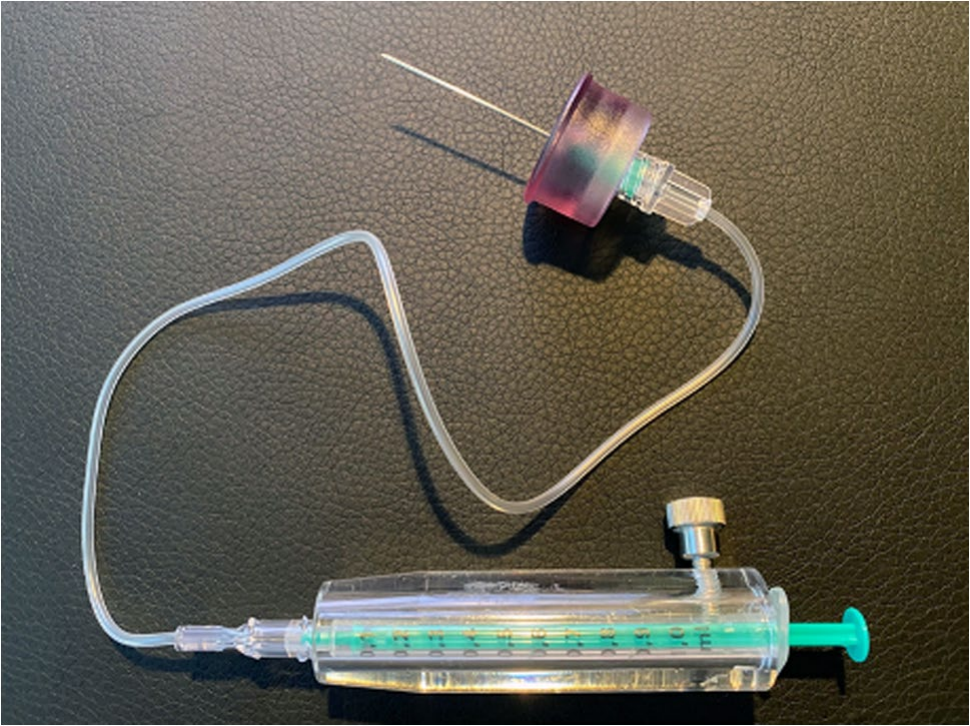
A small flexible tube (volume 350 µl) may be used for difficult injections to avoid displacement of the needle inside the joint when the syringes with a contrast agent, radiocolloid, and glucocorticosteroid are exchanged

- Measurement of the activity (difficult for beta-emitting radionuclides) or calculation of activity by volume based on specific activity given on the vial.
- Use of a suitable syringe and shielding device depending on the radionuclide used (e.g., plexiglass) (Fig. 2). Pliers and tweezers can be used as gripping tools.
- If a difficult procedure is anticipated, a short flexible tube may be used to prevent movement of the needle (Fig. 3).

### General and joint-specific implementation

Intraarticular punctures should be based on published guidelines and use the anatomically most favorable access routes [119, 120]. Efficiently performing RSO contributes to minimizing irradiation exposure to the physician in charge of the procedure and all the medical and paramedical staff. The use of acrylic glass shielding of the syringes will lower the radiation burden of the fingertips during injection. Model-related radiation protection of the fluoroscopy unit has to be assured.

The following recommendations apply:

- Ultrasound or arthrography with image documentation.
- Puncture of a possible joint effusion.
- The radionuclide injection is only to be carried out after the intraarticular position of the needle has been confirmed. This usually requires fluoroscopy, if necessary, after the injection of contrast medium or using ultrasound. Thorough knowledge of anatomical landmarks helps to carry out the procedure with a minimum of pain for the patient.
- There is no general recommendation to use local anesthetics; this depends on the joint to be treated and the sensitivity of the individual patient.
- An important issue for the knee joint should be noted. If X-ray contrast media are used, they should be free of EDTA that may dissolve the complex binding of the [^90^Y]yttrium colloid [121]. For this reason, RSO of the knee joint is commonly performed without contrast medium injection. However, in case of a “dry joint” with less effusion, a hypertrophic synovium or if intraarticular septae are visible, the use of contrast agents may be helpful to assure correct needle placement to achieve a homogeneous distribution of the injected radionuclide.
- In case of RSO using [^169^Er]erbium colloid, the same effect of EDTA-containing contrast media was described and thus, the amount of intraarticularly injected contrast agents during RSO should be as low as possible [122].
- The injection needle should be rinsed with physiological saline or a glucocorticosteroid solution (see below) when treating large- and medium-sized joints to improve the intraarticular radionuclide distribution. Also, it avoids carryover of any activity into the puncture channel.
- Subsequent injection of a glucocorticosteroid: preferably triamcinolone hexacetonide, triamcinolone acetonide, or betamethasone. The combination of [^186^Re]rhenium sulfide with a microcrystalline sustained-release glucocorticosteroid should be avoided in the hip due to the possibility of femoral head necrosis after local injection, regardless of the preparation used.
- After removing the injection needle, cover the injection site with a sterile wound dressing.

#### Joint-specific comments

##### Knee joint

The needle’s intraarticular positioning is checked either by the presence of a synovial effusion, even if minimal, before any injection, or by a small intraarticular injection of contrast agent in the absence of effusion. A paraarticular injection of [^90^Y]yttrium colloid could lead to severe complications such as skin atrophy or even soft tissue necrosis.

The superolateral approach with puncture of the suprapatellar recess is preferred for the knee, allowing the removal of synovial fluid even if it is present in small amounts. Other approaches are possible as well, depending primarily on the experience and the habit of the therapist. The internal subpatellar route is known to be more painful. Bent knee punctures decrease the chances of removing synovial fluid, as the effusion may be pushed into the knee’s posterior compartment. Ultrasound guidance is recommended (Fig. 1B). The landmarks are by palpation: the patella must be held between the thumb and the index finger and its external lateral edge must be marked. A 50-mm or 40-mm, 21 Gauge needle is mounted on an empty syringe to aspirate a possible effusion. By subluxation of the patella, one can slide the needle under the skin and orient it to place it under or directly proximal to the patella’s upper part, simultaneously looking for liquid by gently withdrawing the syringe while aspirating. Using an applicator like shown in Fig. 1A makes puncture and aspiration of joint fluid easier and allows a single-handed procedure with the hand using the ultrasound device.

After correct needle placement, the syringe replacements (aspiration syringe, radionuclide syringe, and corticosteroid syringe) must be done by keeping the needle in place; it must be held during the whole maneuver by a hand resting on the knee. A 3-way valve may be used to allow the injection of the glucocorticosteroid derivative without changing the needle’s position. This valve may also be used for all other joints except the small joints of the hands or feet; however, it is not generally recommended. The injection is slow and painless.

##### Wrist and finger/toe joints

Aspiration of synovial fluid is very uncommon in these joints. Joint puncture should be performed under fluoroscopy control. The simplicity and safety of ultrasonography to find an intraarticular effusion, even in small joints, could, in the future, modify the conditions for carrying out RSO.

The most common approach for the wrist joint is to put a 25 Gauge 25-mm length needle from dorsal into the cruciate fossa (radio-scaphoid-lunate) perpendicular to the skin. This is the entry point, but the radionuclide will most often diffuse throughout the wrist. Indeed, the different compartments of the wrist are most often in communication following the degeneration and rupture of the various ligaments caused by the synovitis.

To prevent the reflux of the different products injected through the puncture port, we propose to apply skin traction when the needle is inserted by giving it a bayonet route. Thus, the radiocolloid will be strictly injected intraarticularly.

The injection in the metacarpophalangeal joint is done with a shorter needle of 25 Gauge and 16 mm. The same needle will be used for the small joints of the fingers to inject [^169^Er]erbium colloid. This is relatively easy by exerting traction in the axis of the finger one can, most often, open the joint and locate the puncture point.

**Fig. 4:**
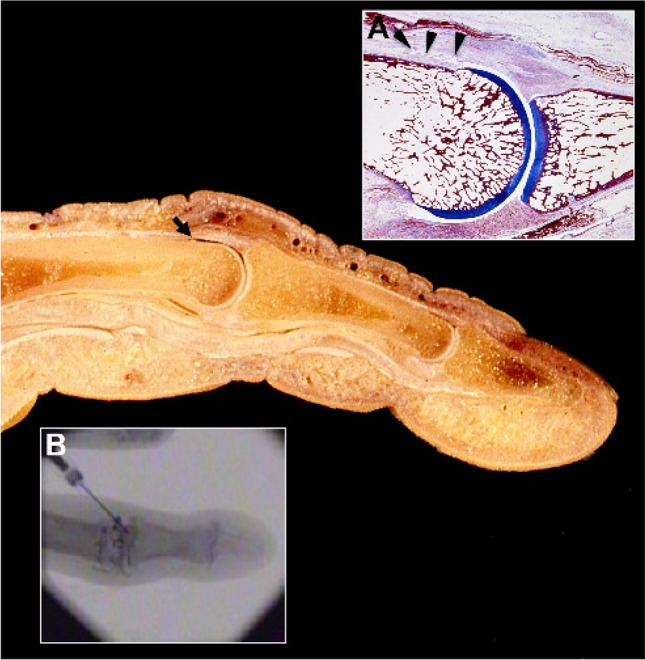
Dorsal recess of a finger joint on a macroscopic anatomy specimen (arrow). Inlay (A) shows the histology of the dorsal recess in a proximal interphalangeal joint (arrow heads) (image by courtesy of Prof. Dr. Bernhard Tillmann, Kiel, Germany) and inlay (B) illustrates the intraarticular distribution of the injected contrast media

Injection into the proximal and distal interphalangeal joints is more difficult because of the small joint size, sometimes even narrowed by small osteophytes. The position of the skin folds in relation to the joint space is assessed by flexing the finger; the needle is placed opposite to the most proximal part of the joint spacing, knowing that in the fingers, the capsular insertion is more proximal than distal. An excellent alternative access route is to inject the radiocolloid into the dorsal joint recess by needle placement dorsally from proximal to distal while the finger is elongated. It is important to lead the needle by a very small angle to avoid a deep injection touching the joint forming bone. It is easy to inject a considerable volume with a few drops of contrast media, radiocolloid, and additional glucocorticoid using this approach even in joints with a narrow joint space due to degenerative changes (Fig. 4). The same holds true for RSO of the respective toe joints.

Injection into the 1st carpometacarpal joint (= Thumb base joint) is done under fluoroscopy by a direct approach to the joint space between the base of the first metacarpal bone and the trapezium. Sometimes it might be helpful if the patient bends his thumb to an ulnar direction, which “unfolds” the joint cavity.

##### Hip joint

Hip RSO can be done under fluoroscopy or ultrasonography. Because of simplicity, the anterior approach under fluoroscopy is preferred. The patient is lying in a supine position; the feet are in internal rotation. A marker could be placed on the skin on the external two-thirds of an imaginary line that goes from the pubis to the greater trochanter. The presence of the iliac neurovascular bundle is checked by palpation, well within the puncture site. A 21 Gauge 50-mm or 20 Gauge 9–10-mm needle is used, positioned at 90° to the plane of the thigh. After bone contact, the needle is withdrawn by a few millimeters. Fluoroscopy is used to confirm the correct positioning of the needle in the middle of the femoral neck and [^186^Re]rhenium colloid can be injected without pressure and pain.

##### Shoulder joint

For the shoulder joint, namely the glenohumeral cavity, three approaches are preferred: the anterior, the upper, and the posterior approaches. It is preferred to position the patient in a supine position, arm in external rotation, and inject through the anterior route. A marker may be placed on the skin after palpation of the joint space by mobilizing the arm. The needle must be positioned in the lower third of the joint space. A 50-mm or 40-mm, 21 Gauge needle is most often used. The needle penetrates the skin at 90° to the plane of the shoulder. Local anesthesia is sometimes useful because the procedure can be painful, especially in contact with the periosteum. Once in contact with the joint capsule, a small internal rotation of the humeral head may be required to allow the needle to slide into the glenohumeral joint. Sometimes an effusion is found and evacuated. If there is no effusion, the correct positioning of the needle is checked by fluoroscopy. The radiopharmaceutical [^186^Re]rhenium sulfide, followed by a corticosteroid, is injected without pressure.

##### Elbow joint

Puncture is done with the patient sitting in an upright position with the elbow bent to 90° and pronated forearm. Anatomical landmarks are easy to palpate: the olecranon, the lateral humeral epicondyle, and the radial head. The center of this triangle is the point for the arthrocentesis. Using fluoroscopy, the needle (usually 18 Gauge) is inserted in the direction of the radial head.

For all joints mentioned in this paragraph, other approaches are possible of course and will have their advantages and disadvantages. If the needle is placed correctly in the joint cavity, regardless of the injection pathway, the puncture is done properly and RSO could be performed safely.

A lot of different approaches are published in corresponding textbooks of rheumatology and orthopedics. These references should be adhered to, even in other joints not mentioned in this guideline (e.g., sacroiliac joint, temporomandibular joint).

### Modifications in pediatric hemophilia

The selection of nuclides in hemophilia patients is not different from that in other diseases. However, it is not yet clear if the choice of radionuclide affects treatment success [72]. Typically, [^90^Y]yttrium citrate is used for knee joints and [^186^Re]rhenium sulfide for medium-sized joints. Country-specific requirements or approvals must be observed (e.g., [^169^Er]erbium citrate is not approved for a pediatric population in Germany). The selection of radionuclide and the applied activity depend on patient weight and age, joint size, and the thickness of the synovium in children. For knee joints, the procedure could be performed without fluoroscopy solely, while in all other joints, fluoroscopy, and contrast medium arthrography are advisable, especially in case of a narrow joint space or more extensive osseous deformities [55, 123].

Whether [^186^Re]rhenium colloid is to be used for RSO of a knee joint in smaller children should be decided based on the individual case. Despite the reduced depth of penetration compared to yttrium-90, it can be assumed that the same effect can be achieved with less radiation exposition of the joint and the surrounding tissue. However, due to the good long-term experiences in hemophiliac patients, [^90^Y]yttrium colloid could also be considered for treating the knee in the pediatric population [124].

There is no general recommendation for the injected activities in the pediatric population, but it should be individualized based on the patient’s age and weight and the size of the joint. In the published studies, half of the adult activity was either generally applied to children or if the body weight was below 20 kg [125, 126].

An individualized dose concept has not been established yet for synovitis in hemophilia because of the difficult dosimetry due to the variable amount of synovial tissue [50]. Also, there is no consensus on age limitations for performing RSO, although some papers propose a lower limit of 2 years [59] or even 1 year of age [79].

At the time of RSO, the optimal clotting factor replacement levels must be achieved, based on a substitution plan prepared by the hematologist. Because of the increased bleeding risk after RSO, an intensified clotting factor substitution therapy must be continued for at least 3 months [58]. General recommendations for RSO in pediatric patients considering age and target joint are shown in Table 5.

**Table 5:** General recommendations for RSO in pediatric patients considering age and target joint (joint volume, degree of synovitis)

Joint	Recommendations
Knee	Pre-adolescent: [^186^Re]rhenium colloid (activity 50–100 MBq) Adolescent: [^90^Y]yttrium colloid (activity 150–185 MBq)
Ankle	[^186^Re]rhenium colloid (activity 40–75 MBq)
Elbow	[^186^Re]rhenium colloid (activity 30–60 MBq)

Precise knowledge of the age-related X-ray anatomy of the joints and epiphyseal plates is essential for the puncture technique. The ultrasound anatomy of the joints in children should also be known, keeping in mind especially the epiphyseal plates. The bone core of the patella is not visible in small children until the age of 3 to 4 years. Also, the cartilage is thicker than in adults and hypoechoic (black on ultrasound); attention should be made not to confuse it with effusion.

Usually, in children older than 8 years, RSO can be performed without the need for sedation. If there are concerns about pain-memory formation, young children can be treated with NSAIDs. Because of the risk of increased bleeding, the hematologist should be consulted before the treatment. In smaller children or in particular conditions (e.g., when a difficult joint puncture is anticipated, an anxious patient), sedation should be considered beforehand. Sedation in children should only be performed by an anesthesiologist or physician experienced with pediatric emergencies.

### Repeating RSO

RSO can be repeated after 6 months, if a sufficient therapy success has not been achieved after the first application. A visual analog scale (VAS), for example, is useful for evaluating the success of treatment based on subjective pain sensation. If the pain symptoms are not at least 50% lower, the therapy success is not sufficient. The shortest interval for repeating RSO is at least 6 months according to the technical information and instructions for use. However, the approved maximum annual cumulative administered activity of the respective agent must be observed. For example, in Germany, the maximum cumulative activity is limited to 444 MBq for [^90^Y]yttrium colloid, 444 MBq for [^186^Re]rhenium colloid, and 300 MBq for [^169^Er]erbium colloid per year.

### Immobilization

The treated joint should be immobilized and relieved for about 48 h using a rigid splint to prevent movement-related reflux of the activity via the remaining puncture channel with the risk of skin necrosis or an outflow via the lymph channels. Bed rest is not required. In cases where immobilization cannot be ensured, inpatient therapy should be considered. This is especially true for the large- and medium-sized joints of the lower extremities. The immobilization of large- to medium-sized joints of the lower extremity (hip, knee, ankle) may require thromboembolic prophylaxis, especially in patients who are at risk for thromboembolism or if two or more adjacent joints are treated and, thus, immobilized [127, 128].

### Scintigraphy for documentation of the intraarticular distribution of the radiocolloid

**Fig. 5:**
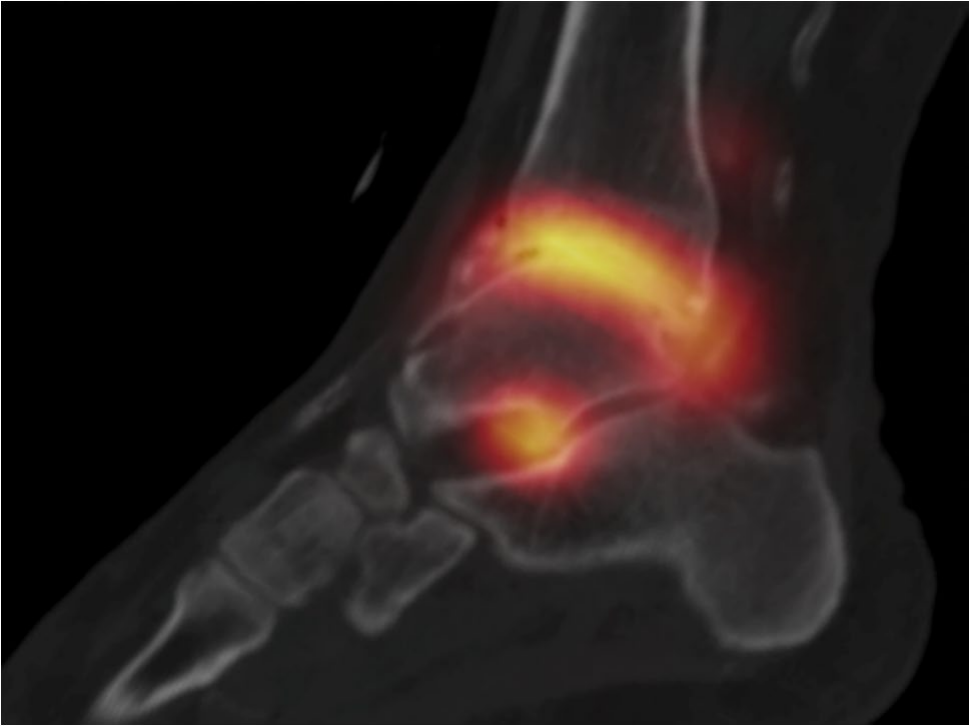
Distribution scan by SPECT/CT 30 min after intraarticular injection of 74 MBq [^186^Re]rhenium sulfide in an ankle joint of a patient with rheumatoid arthritis

After the injection of [^186^Re]rhenium colloid (gamma component) and [^90^Y]yttrium colloid (Bremsstrahlung), the intraarticular radionuclide distribution should be documented immediately by scintigraphy, in case of [^90^Y]yttrium colloid also by PET/CT. Moreover, any extra-articular radionuclide distribution in case of a paraarticular (false) injection can be excluded by this procedure. Imaging 48 h after RSO could be helpful to detect lymphatic drainage from the treated joint (Fig. 5).

### Patient follow-up

It is recommended to review patients 4 to 6 days after the procedure to evaluate possible early side effects. Further multidisciplinary clinical follow-up is recommended 4 to 6 months after treatment [129].

### Radiation protection of staff

**Fig. 6:**
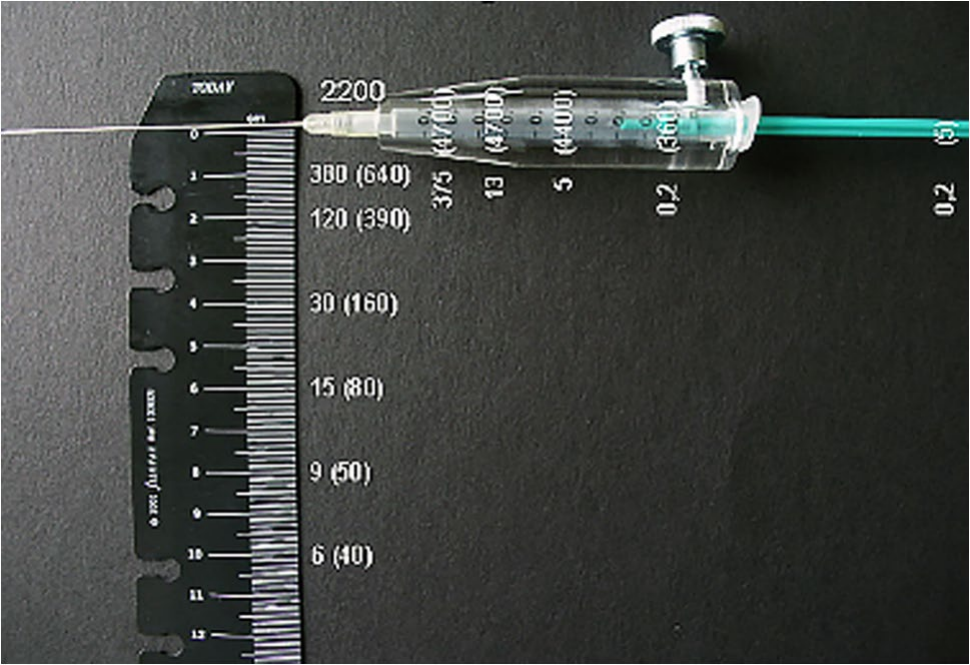
Dose rate at the surface of a plexiglass shielding of a syringe filled with 185 MBq yttrium-90 in different distances from the neck of the hollow needle. Values in parentheses show the dose rates without shielding. (reproduced with permission of the Bundesamt f. Strahlenschutz BfS)

Concerning radiation protection, the special properties of beta emitters must be taken into account during the application, which due to their short range can cause a high surface dose. The surface personal dose H_p_(0.07), representing the equivalent dose at a depth of 0.07 mm in the body at the position of the partial body dosimeter, is particularly relevant for radiation protection. As known, beta radiation can be shielded well by materials with a low atomic number, for example, by syringe shields made of acrylic glass (Plexiglas®, PMMA). As an approximation, the thickness $D$ required for shielding can be calculated from the maximum beta energy of the radionuclide using the following rule of thumb: $D\left[{\text{cm}}\right]={~}^{{E}_{max}[{\text{MeV}}]}\!\left/\!\!{~}_{2}\right.$. So, beta radiation from yttrium-90 is almost completely shielded with a shield made of approximately 1 cm of acrylic glass, documented by dose measurements done by the German Federal Office for Radiation Protection (BfS), shown in Fig. 6.

In addition to the consistent use of such shields, distance-increasing devices must be used to prepare the syringes and apply the radiopharmaceutical. For this purpose, it is recommended to use additional holding forceps or tweezers to fix the cannula [130] or to use a disposable polycarbonate ring (Makrolon®) during application (see Fig. 2B). Adequate radiation protection during fluoroscopy also has to be considered, including protective X-ray lead aprons. Likewise, positioning the X-ray tube under the table to minimize stray radiation is recommended. The hands of the physician performing the procedure must not be located in the radiation beam during fluoroscopy.

All syringes prepared for one RSO setting must be stored in a plastic box with adequate shielding before use. The waste should be collected with clear-cut separation between the three radionuclides used. Their different half-lives determine the clearance of the waste for its final disposal according to national regulations.

## Dosimetric considerations

### Absorbed dose to the target region

The options for individual determination of the absorbed dose to the treated synovium and cartilage are limited. A 2-D Monte Carlo model for the synovial joint has been developed for calculating depth-dose profiles for source activity distributed in the synovial fluid [131]. The absorbed dose from this model is strongly dependent on the synovial surface area, and it assumes a uniform thickness of 0.74 mm (0.5 mm fluid and 0.24 mm synovial lining) for the source distribution. The absorbed doses to the synovium estimated using this dosimetry model were reported to be 2.3–4.3 Gy with [^186^Re]rhenium and 9 Gy with [^169^Er]erbium [132]. This value is much lower than the absorbed dose of approximately 100 Gy in the source volume reported for [^90^Y]yttrium-based RSO to reach similar effects as surgical synovectomy [3]. The dosimetry model to calculate the mean absorbed dose to the source volume is most often based on the sphere models within most dosimetry software [133, 134]. An example of the absorbed doses to the spheres is indicated in Table 6, assuming no leakage from the injection site. Due to the difficulty of individualized dosimetry because of the large difference in synovial thickness, the administered activities in RSO are, in most cases, based on empiric knowledge.

**Table 6:** Absorbed dose per administered activity (D/IA) of respectively [^90^Y]yttrium, [^186^Re]rhenium, and [^169^Er]erbium in unit-density spheres, assuming permanent encapsulation in the spheres, based on output from Olinda/EXM v 2.1 software

**Sphere mass [g]**	**D/IA [Gy/MBq** [^90^Y]yttrium**]**	**D/IA [Gy/MBq** [^186^Re]rhenium**]**	**D/IA [Gy/MBq** [^169^Er]erbium**]**
0.01	1136	1674	1031
0.1	260	212	103
0.5	70.0	45.3	20.7
1	37.9	23.0	10.4
2	20.1	11.7	5.20
4	10.5	5.91	2.60
6	7.17	3.97	1.73
8	5.46	2.99	1.30
10	4.40	2.39	1.04
20	2.25	1.20	0.52
40	1.15	0.61	0.26
60	0.78	0.40	0.17
80	0.59	0.30	0.13
100	0.47	0.24	0.10
300	0.16	0.08	0.03
400	0.12	0.06	0.03
500	0.10	0.05	0.02

The potential of using post-therapy imaging with [^90^Y]yttrium Bremsstrahlung SPECT/CT and PET/CT has been demonstrated for verifying uniform distribution and target dosimetry [135, 136]. This verification is of great importance to have a timely identification of activity outside the joint, either by leakage or by misplacement of the needle.

### Factors influencing dosimetry

#### Resting/splinting

Studies comparing inpatient care using bed rest or outpatient care using a rigid splint for immobilizing the treated joint have found no difference in leakage risk [137–139]. The use of a semi-rigid splint in outpatient care resulted in a doubling of the lymph-node uptake [138]. Using biological dosimetry, the calculated dose was five times higher without immobilization compared to bed rest [140]. The duration of immobilization in the studies mentioned above ranged from 48 to 72 h. Therefore, the treated joint should be immobilized using a rigid splint. If this is not possible (e.g., hip), patients could be immobilized by bed rest to avoid a significant leakage.

#### Leakage

##### [^90^Y]yttrium colloid

In case of strict immobilization of the knee joint, a mean total activity leakage of 1.8% (± SD range: 0.45–4.78%) [137] and 1.9% (range: 0–13%) [141], respectively, was reported. When injecting 200 MBq, this leakage results in mean organ doses of 619 mGy (16th and 84th percentile: 154 mGy – 1644 mGy) for the lymph nodes and 62 mGy (16th and 84th percentile: 15 mGy – 165 mGy) for the liver and spleen. The mean effective dose was 37 mSv (16th and 84th percentile: 9 mSv – 99 mSv). The dose to the gonads as a combination of the doses resulting from activity leakage and Bremsstrahlung from the activity in the knee was estimated to be 0.1 mGy (16th and 84th percentile: 0.05 mGy – 0.18 mGy) in women and 0.2 mGy (16th and 84th percentile: 0.10 mGy – 0.38 mGy) in men [137].

In comparison, Gratz et al. [31] calculated doses of 155 ± 94 mGy for the whole body, 265 ± 133 mGy for the liver, 119 ± 101 mGy for the spleen, and 671 ± 332 mGy for the kidneys. The actual biological effect of leakage can be assessed by measuring the increase in chromosomal aberrations, and a clear correlation has been reported between both parameters [140, 142, 143]. Indeed, an increase in chromosomal aberrations can be expected with leakages of 5% and higher [144]. If strict immobilization is used, leakage is considerably lower and accordingly, an increase of chromosomal aberrations as a sign of a biological radiation effect had not been demonstrated [145].

##### [^186^Re]rhenium colloid: 

A general conclusion based on the data on activity leakage and radiation exposure in RSO using [^186^Re]rhenium sulfide is impossible due to high variability in injected joints, activities, and immobilization strategies. The available data mainly measured activity leakage from RSO of the ankle and the upper extremity joints. The total mean leakage ranged from 2.5 to 3.9% [141, 146, 147]. Whether joint-related differences exist has not yet been investigated. Other authors reported a whole-body dose of 53 ± 27 mGy by measuring activity leakage [31], and an effective dose of 27 ± 5 mSv when measuring blood activity [148]. The organ doses were calculated to be 100 ± 80 mGy for the liver, 203 ± 229 mGy for the spleen, 94 ± 113 mGy for the kidney, and 26 Gy (range 0–189) to the lymph nodes (when joint immobilization was not possible), respectively [141]. After RSO of the wrist, no significant increase of dicentric chromosomes could be found [146].

##### [^169^Er]erbium colloid

There are limited clinical data on activity leakage. A study including 7 patients found a leakage of 0.11 ± 0.3% [93]. The whole-body radiation dose was calculated to be 4.1 ± 2.5 mGy and < 1 mGy/30 MBq, respectively [31, 148]. An increase in chromosomal aberrations could not be found [146, 148].

#### Baker’s cyst

When performing MRI of the knee joint, the prevalence of Baker’s cyst is between 5 and 19% [149, 150], and with ultrasound, a prevalence of 25% was reported [151]. A rupture of Baker’s cyst shortly after RSO can occur due to increased pressure in the cyst and is considered problematic. One possible cause may be the existence of a unidirectional valve mechanism in Baker’s cyst. However, anatomical and clinical studies have shown that a unidirectional valve mechanism is very unlikely and irrelevant as a risk factor for cyst rupture [152]. Especially, with strict immobilization, no activity accumulation in Baker’s cyst over 46 h after RSO was found [153]. In addition, the calculated activity in the fluid 48 h after RSO based on a puncture of a knee joint with Baker’s cyst is less than 40 kBq/ml. Therefore, significant damage to the popliteal and calf tissue in case of rupture should not be expected [152].

On the other hand, a positive effect of RSO on Baker’s cysts has been reported. A recent study, which examined the volume reduction of Baker’s cysts in 102 RSO procedures, found a volume reduction in the range of 50% up to 7 months after RSO. In some cases, the effect could be demonstrated up to an average of 36 months after RSO [154].

In conclusion, only a ruptured Baker’s cyst at the time of RSO is a relevant contraindication. Furthermore, the positive effect of RSO on the volume of existing Baker’s cysts warrants the inclusion of this technique into a multimodal treatment approach for this condition.

## Side effects and complications

RSO has a very low rate of side effects and complications when appropriately performed by experienced clinicians. Fischer et al. recently published data on the prevalence of adverse events (AEs) with radiopharmaceuticals from 1990 to 2011 and reported 3.3 AEs/100.000 treated joints [6]. Data up to 2019 show a value of 4.5/100.000 and are based on AEs reported to the companies distributing the radiocolloids approved for radiosynoviorthesis (Table 7). The number of treated joints was calculated from the total amount of activity supplied throughout Europe, and the routinely applied activities per joint minus 10% compensating for loss.

**Table 7:** The number of adverse events after radiosynoviorthesis

Radiopharmaceutical, time interval	Number of treated joints	Serious AEs (data in brackets from [155])	Non-serious AEs
[^90^Y]yttrium citrate, 1990–2019	± 452.000	35 (19)	53
[^90^Y]yttrium silicate, 1994–2003	± 70.000	3	2
[^186^Re]rhenium sulfide, 1990–2019	± 415.000	21 (3)	29
[^169^Er]erbium citrate, 1990–2019	± 583.000	10	17
Total	± 1.522.000	69 (22)	99
Incidence		4.5/100.000 (6.0/100.000)	

Intraarticular infections (*n* = 18) accounted for most serious AEs and are related to the procedure and not to the radiocolloids themselves (see separate discussion below). Other serious AEs were radionecrosis, lung embolism after immobilization, or an anaphylactic reaction. Examples of non-serious AEs are transient redness or pain at the injection site, flush symptoms from intraarticular steroid co-injection, or transient and self-limiting radiation synovitis, with recurring joint effusion after RSO.

Kisielinsky et al. analyzed patients who had surgical interventions after RSO [156]. It should be noted that many of the patients in this study were treated with RSO more than two times or had severe degenerative joint destruction with a Kellgren-Lawrence stage of III or IV. Also, the number and type of treatments before RSO was not reported in this paper, especially prior intraarticular applications of glucocorticosteroids. A high rate of osteonecroses (in 22 out of 93 patients) was observed after RSO, but no information was given on pre-therapeutic imaging. Taking into account that more than 10% of avascular osteonecroses occur in patients with hip osteoarthritis and that intraarticular corticosteroids or other factors like diabetes or osteoporosis (information not provided by the authors) may lead to osteonecrosis even before RSO, it is difficult to justify that these cases are attributed solely to RSO by these authors.

Following the WHO definitions, an adverse event or experience is defined as “any untoward medical occurrence that may be present during treatment with a medicine but which does not necessarily have a causal relationship with the treatment.” Every healthcare professional, even every person working in a medical institution, is obliged to report adverse events to the pharmaceutical company distributing the respective compound.

Most of these side effects and complications are preventable by a skillful injection technique under aseptic conditions and if the correct radionuclide in an appropriate activity is applied for therapy. Recognition of these potential side effects is essential to establish a proper therapeutic strategy and avoid unnecessary treatment.

### Radiation synovitis

A temporary increase in joint pain and swelling due to radiation-induced synovitis and joint effusion 6 to 48 h after treatment may be observed [157]. Lymphedema or fever may occur in rare cases. These symptoms are usually self-limiting without further intervention and can be treated simply by cooling the joint with ice packs or, if necessary, with anti-inflammatory drugs. A flare in inflammatory symptoms (radiation synovitis) after RSO may be considered a natural course of the treatment and a clinical manifestation of rapid and extensive synovial tissue necrosis. It is more common after RSO using radiopharmaceuticals with high energy (e.g., [^90^Y]yttrium colloid). For large joints, a small amount of glucocorticosteroid can be injected during RSO to reduce inflammation induced by radiation [158]. In addition to reducing inflammation, glucocorticosteroids also decrease systemic radioisotope leakage through dilated capillaries of the synovium. For RSO of medium and small joints, some authors advise intraarticular administration of a local anesthetic instead of glucocorticosteroids [159, 160]. However, no published studies have shown significant differences or advantages between these two options.

### Infection

Joint infection is one of the more severe complications which is not related to the radiopharmaceutical agent itself but might occur from any, even diagnostic joint puncture by microbial spreading. After intraarticular injection, the risk of infection is generally assumed to be very low and ranges from 1:3000 down to 1:100.000 [161], but the data concerning joint infections after RSO are scarce [155, 156, 162]. Also, the individual risk for an intraarticular infection depends on several predisposing factors (e.g., diabetes, systemic inflammatory disease, immune status, concomitant medications, joint arthroplasty). When clinical signs of intraarticular infection after RSO develop, immediate fluid aspiration and bacterial culture must be performed. If an initial oral antibiotic treatment does not improve the situation significantly within 24 to 48 h, the infection should be treated by joint lavage or endoscopy together with the local application of intraarticular antibiotics [163].

### Radiation tissue damage

Superficial skin and needle track ulcerations caused by radiation may occur if the radionuclide leaks from the needle during retraction or through the puncture channel from the joint after injection [164–167]. These complications can easily be avoided by flushing the needle (with steroid or saline) and compression of the injection site after the radionuclide injection. Skin discoloration, thickening, blisters, or formation of a small scar at the injection site are common signs of radiation tissue damage but usually have no clinical consequence. The patient may rarely experience a burning sensation in the injection area, most likely due to irritation of small nerve fibers [168]. The time elapsed from the procedure to the ulcer appearance varied from 3–4 weeks to 8 months.

More severe and more profound skin necrosis appears to be an infrequent complication after radiosynoviorthesis. However, in 2006, a German survey performed with 260 nuclear medicine physicians and 20 medical liability insurances detected 28 cases of skin necrosis [156]. Furthermore, the possibility that this side effect may be underreported cannot be ruled out. Possible treatments include hyperbaric oxygen therapy, surgical debridement, and autologous skin transplantation. If necrosis occurs, surgery should be delayed because, as in the case of beneficial effects of RSO, it may take some time for the damage to reach its full extent [169]. [^90^Y]yttrium is the most potent radionuclide to induce tissue damage, possibly even full-thickness skin necrosis [170]. Beta radiation burns after [^186^Re]rhenium colloid are much more limited, usually self-healing within 3–4 weeks, but in severe cases, hyperbaric oxygen treatment is helpful [171–173]. [^169^Er]erbium colloid, with its low energy and small tissue penetration, is unlikely to cause more profound necrosis, and beta radiation burns associated with its use require only conservative treatment [3].

However, long-term monitoring after dermal radiation injury appears advisable as patients with radiation exposure also have an increased risk of secondary malignancy, particularly non-melanoma skin cancer.

#### Radionecrosis of the juxta-articular soft tissue

Necrosis of periarticular tissue is the most severe local complication in RSO and is caused by the accidental paraarticular injection of the radionuclide or leakage. During the actual joint puncture, all measures should be taken to ensure the correct position of the needle tip and complete intraarticular injection. Extra-articular injection or leakage can cause extensive damage to healthy tissues since beta radiation can induce necrosis of the synovial membrane and any other soft tissue. Only a few cases related to periarticular necrosis have been reported in the literature [169, 174, 175]. A distribution scan acquired with a gamma camera after radionuclide injection helps to verify successful intraarticular injection and proper distribution within the joint. When periarticular tissue necrosis occurs, patients usually develop pain and functional impairment. In cases of radionecrosis or paraarticular injection, no reliable recommendations exist owing to limited experience. One alternative would be to wait and observe until demarked necrotic tissue can be resected. Another alternative would be to perform surgery immediately to remove as much of the para-injected activity as possible by flushing the tissue and resecting radioactive tissue around the injection site. However, precautions have to be taken to protect the surgical team against radiation from the contaminated tissue. Since there is no experience in this field, any individual case must be discussed between the surgeon and the nuclear medicine physician.

#### Influence on the cartilage and bone

Healthy intra- and extra-articular tissues such as the cartilage and bone may be exposed to the radioactivity injected into the joint. Although mature cartilage is considered resistant to radioactivity, even minor injury to articular cartilage remains a concern for RSO, especially in the pediatric population. However, most studies do not support these concerns, ruling out a direct link between RSO and chondrocyte damage or acceleration of osteoarthritis [157]. Moreover, RSO may lead to a significant decrease in inflammatory cells, levels of proteolytic enzymes, and metalloproteinases harmful to the cartilage, to some extent preventing further joint damage. In animal models, transient radiation effects were observed only in young, growing cartilage [176, 177]. Additionally, synovial damage, subsequent joint inflammation, and fibrosis may also contribute to further articular cartilage damage after intraarticular radiocolloid injection and, thus, is not imperatively linked to any radiation effects [178].

Although not as large as that to cartilage, the absorbed dose to the bone surface and red bone marrow is also important in RSO. The bone surface dose was described as being 25% of the synovial surface dose with [^90^Y]yttrium, 4% with [^186^Re]rhenium, and negligible for [^169^Er]erbium colloid [131]. For the bone surface, a maximum dose of 18 Gy was calculated for 185 MBq of [^90^Y]yttrium, which is not considered sufficient to cause significant bone damage or necrosis. On the other hand, recent studies suggest that in patients with end-stage osteoarthritis and extensive cartilage erosion, beta radiation may cause avascular necrosis of the exposed subchondral bone [179]. While the immediate sequelae of this condition may be clinically silent, avascular osteonecrosis may compromise future surgical joint arthroplasty procedures. When there are already bony changes on X-rays, it is better to avoid RSO considering its efficacy and safety, as discussed earlier. The dose to the bone marrow in large- or mid-sized joints is considered negligible because the distance to the radiation source is greater than the mean tissue penetration of radionuclides used for RSO [180].

An influence of RSO on the epiphyseal growth plates in children has not been described.

### Thromboembolic complications

Thromboembolic complications are not specific for RSO but may occur because of the mandatory immobilization of the treated joint. Risk factors for thromboembolic complications after RSO of lower limb joints include older age, reduced mobility, varicose veins, or pre-existing coagulation disorders. Provided there are no pre-existing risk factors, the risk of a thromboembolic complication must be carefully weighed against possible side effects from anticoagulation therapy. Therefore, thromboembolic prophylaxis is not generally recommended. It is recommended after immobilization only in patients after RSO of two adjacent joints of the lower limb and patients with at least two risk factors for thromboembolic complications [163].

### Genotoxic effect and cancer risk

The effective doses for RSO given in dosimetry studies seem to be in the low-dose range when there is no major leakage [146, 181]. Radiocolloid particles that leak out of treated joints could accumulate in the regional lymph nodes and the liver. The absorbed dose to neighboring lymph nodes could be higher and in the deterministic range of radiation effects depending upon the severity of leakage and patient characteristics [182].

There have been two cases of acute lymphocytic leukemia reported in hemophilia patients receiving phosphorus-32. However, due to the short interval between radiation exposure and malignancy, the causal relationship between phosphorus-32 and leukemia development cannot be established in these patients. Moreover, both children had other autoimmune disorders [7, 183]. While the current literature is limited regarding the long-term risks of cancer, a recent retrospective study including 2412 adult patients treated with RSO by using [^32^P]phosphorus or [^90^Y]yttrium colloid revealed no increase in cancer risk compared to the general population [7]. Similar results were reported by Vuorela et al. [184]. That study compared the medical records of 1228 rheumatoid arthritis patients with and without RSO; no evidence of increased cancer incidence was found following intraarticular treatment with [^90^Y]yttrium colloid.

An increased risk of cancer after RSO with radiocolloids has not been reported. Many studies reported cytogenetic analyses, such as chromosomal aberration analysis, micronuclei, and sister chromatid exchange, to indicate radiation-induced cytogenetic damage in hemophilic children undergoing RSO with [^90^Y]yttrium or [^186^Re]rhenium colloid [145, 185–188]. These studies indicate that high radiation doses, which would induce genotoxic effects, are not obtained in peripheral blood lymphocytes in children after RSO. Intraarticular treatment using radioactive colloids has been performed for more than half a century. The extensive clinical experience and the lack of any well-documented secondary malignancies resulting from RSO suggest a very low and acceptable risk compared with the benefit for the patient.

### Bleeding risk in hemophilia

There is a risk of bleeding in patients with hemophilia due to puncture, which is very small if there is sufficient factor substitution [58]. It is essential to provide sufficient factors up to 3 months after RSO, according to the healing of the synovitis. During this time, an increased bleeding tendency can be assumed [58].

## Place of RSO compared to alternative treatment strategies

The key feature of arthritic conditions is primarily the presence of synovitis. Joint involvement and distribution depend on the type of arthritis. The treatment options for patients with arthritis have increased considerably within the last decades, especially with the introduction of biological disease-modifying anti-rheumatic drugs (bDMARDs) in the late 1990s. Recommended treatment strategies include early and aggressive treatment with mono- or combination therapy using first conventional synthetic DMARDS followed by bDMARDs. The clinical treatment goal is to obtain rapid disease control and suppress inflammation, thereby preventing pain and joint destruction and improving daily function and quality of life. This strategy was first outlined in the European League against Rheumatism (EULAR) 2010 treat-to-target recommendations where the treatment target was specified as clinical remission or at least low disease activity [18]. Disease activity is assessed by using composite scores that include joint assessment for swelling and tenderness, patient- and physician assessment, and C-reactive protein for evaluating the degree of disease activity.

The use of intraarticular glucocorticosteroid injection is a standard treatment in rheumatological practice combined with DMARDs in the early phase of the disease and as a treatment for joints with flare. In a randomized controlled trial of early RA patients, aggressive step-up treatment with methotrexate combined with intraarticular betamethasone (in a maximum of 4 joints per visit) produced rapid and effective disease control [189]. Repeated steroid injections were well tolerated and safe [190]. Frequently used glucocorticosteroids used are triamcinolone and methylprednisolone.

Despite new drugs and aggressive treatment strategies, including the treat-to-target approach, some patients have persistent synovitis in a single or a few joints where surgical synovectomy or RSO may be indicated. RSO appears to perform better in RA patients than in OA patients [5, 90, 94, 191] where the success rate for the reduction in pain, joint swelling, and tenderness was higher in RA than in OA patients independent of the type of joint. The lowest effect has been reported for undifferentiated arthritis [191].

While many studies have investigated the effect of RSO compared with intraarticular GC injection, their findings have been conflicting due to heterogeneity between trials. There are major differences in administering the radionuclide with or without GC, variability in the type of GC, and very important differences in the patient inclusion criteria. Furthermore, differences in clinical outcomes, including the lack of validated methods for measuring the clinical effect of RSO, make a comparison between studies difficult. Given that synovitis refractory to intraarticular GC treatment is a common clinical problem in rheumatology, RSO provides an additional treatment alternative to surgical synovectomy. Further studies are needed to establish wherein the treatment algorithm of RA RSO has its best place.

For hemophilic patients, RSO has a clearly defined place in the treatment algorithm with persistent chronic synovitis after intensified factor therapy over 6 months respectively 3 joint bleedings per year, as supported by other guidelines [4].

## Liability statement

This guideline summarizes the views of the EANM bone and joint committee. It reflects recommendations for which the EANM cannot be held responsible. The recommendations should be taken into context of good practice of nuclear medicine and do not substitute for national and international legal or regulatory provisions.
